# Measuring episodic memory and mental time travel: crossing the species gap

**DOI:** 10.1098/rstb.2023.0406

**Published:** 2024-09-16

**Authors:** Eli Collaro, Robert A. Barton, James A. Ainge, Alexander Easton

**Affiliations:** ^1^ Department of Anthropology, Durham University, Durham, UK; ^2^ School of Psychology and Neuroscience, University of St Andrews, St Andrews, UK; ^3^ Department of Psychology, Durham University, Durham, UK

**Keywords:** rodent, human, scene construction, mental projection, 4E cognition

## Abstract

Mental time travel is the projection of the mind into the past or future, and relates to experiential aspects of episodic memory, and episodic future thinking. Framing episodic memory and future thinking in this way causes a challenge when studying memory in animals, where demonstration of this mental projection is prevented by the absence of language. However, there is good evidence that non-human animals pass tests of episodic memory that are based on behavioural criteria, meaning a better understanding needs to be had of the relationship between episodic memory and mental time travel. We argue that mental time travel and episodic memory are not synonymous, and that mental time travel is neither a requirement of, nor an irrelevance to, episodic memory. Mental time travel can allow improved behavioural choices based on episodic memory, and work in all species (including humans) should include careful consideration of the behavioural outputs being measured.

This article is part of the theme issue ‘Elements of episodic memory: lessons from 40 years of research’.

## 1. Introduction

Mental time travel (MTT) relates to the experiential aspects of episodic memory or episodic future thinking; the projection of a personal perspective to a specific remembered past or imagined future [1]. Experiential aspects of memory are usually disentangled and related to others by being narrativized—a process that, in most cases, involves translating these experiences into language. Therefore, accessing others’ inner experience requires a level of language sophistication, as well as a working assumption that the experiences described map onto one’s own understanding of them (to make them interpretable). Inevitably, these approaches push us towards framing MTT as a uniquely human experience [2], or to formulate assumptions about the cognitive process involved by imagining other species’ experiences and minds as mirroring our own (e.g. [3]). This poses a serious dilemma to efforts to translate work from the clinic to the bedside around memory loss in conditions such as Alzheimer’s disease. We need, in animals, to be able to accurately model the cognitive processes that fail in disease [4], and in MTT, we have a proposed cognitive process that is critically important in the most prevalent and debilitating disorder of memory with no apparent equivalent in animal models. This severely limits our ability to test models of Alzheimer’s disease and produce pharmacological interventions.

Nevertheless, the gradualist nature of evolution makes it highly unlikely that a significant cognitive mechanism has arisen in humans yet is completely absent and has no basis at all in the brain mechanisms and cognitive processes found in other species. Adaptive specializations do not appear miraculously from nothing in the brain over a single generation or speciation event, but are built from pre-existing structures that are modified, re-used and re-purposed [5,6]. The human experience of MTT is therefore likely to have had a long evolutionary history, most of which was shared in common with other living species. Indeed, when looking at other areas of cognition, it is relatively uncontroversial in comparative psychology that non-human species show evidence of sophisticated cognitive skills, such as planning (e.g. [7]), complex social understanding (e.g. [8,9]) and some foundational aspects of language and related processes involving hierarchically organized sequences (e.g. [10,11]). There is even some evidence for more introspective aspects of cognition around memory (e.g. [12]) and theory of mind [13]. Yet, there remains some resistance to accepting that MTT might not be uniquely human (e.g. [2]).

## 2. The importance of behaviour as the output of a cognitive process

Without the self-reporting enabled by language, our route to accessing such cognitive processes is through behaviour and yet behaviour requires interpretation, and the link between behaviour and the cognitive process requires some assumptions to be made. Branchi [14] outlines the unique privilege held by behaviour; acting as an interface between the environment and the brain. Critically, from this perspective, behaviour is not only the way in which the brain interacts with the environment, but also the way in which the environment interacts with the brain. The brain (and the cognitive processes it mediates) can cue a behavioural response, but aspects of the environment can also trigger a particular behaviour.

In our everyday life, MTT can be spontaneously generated, either through internal cues (e.g. I feel hungry, so I start thinking about what I might eat for dinner) or the environment (e.g. I walk past my old school and remember a particular event that happened there). Critically, these two types of cues can interact. If I am hungry and so I think about what I want to eat for dinner, the environment (e.g. what is available in the fridge) affects what meals I can realistically imagine constructing in my immediate future (‘ah, I could mix those eggs with that cheese and those mushrooms and cook them in that pan’). Behavioural studies of MTT, then, should allow us to observe a behaviour that is flexible and responsive to both internal and external conditions, based on personally experienced events. Observing a hungry person passing by a plate full of toast to choose a different food might be considered a behavioural measure of their memory that they have recently eaten a lot of toast. The behaviour is internally generated (a response to being hungry) and limited by external factors (what food is available) yet is also determined by a personally experienced event (recently eating toast). None of this should be predictable at the time of the event being experienced [15], so the response is a flexible one in that the eating of toast is not specifically encoded in the knowledge it would be useful later. If toast were the only option available, they might produce variety by instead choosing to add peanut butter, rather than the jam used previously.

Using behaviour in this kind of way to assess episodic memory (or, more cautiously and in the authors’ words ‘episodic-like memory’) in animals was first done by Clayton & Dickinson [16] in scrub jays. The birds cached two types of food, one in each of two caching trials. One food type (mealworms) would be cached in one area of a storage tray, while the other food type (peanuts) was stored in a different area of the same tray. Importantly, the two food types had differing values from the scrub jays. While both foods were palatable, mealworms were the food of preference for the birds. However, the environment also constrained the task, while mealworms were higher value than the peanuts, over time they decayed and became unpalatable. Therefore, after short delays (4 h), the mealworms were the higher value food stuff, but after lengthy delays (124 h), the peanuts were the higher value reward. During a test trial, then, the hungry birds wished to recover food (internal drive for memory), but the environment gave a value to the food which was unpredictable at the point at which the birds stored the food (lacking cues as to whether they would be tested at a long or short delay). Birds were therefore able to use the internal cue (hunger) and the external environment (how long since the food was cached) to flexibly adjust their behaviour according to where they had stored the particular food being retrieved. This behavioural operationalization of episodic memory became known more neutrally (with respect to internal representations) as what–where–when memory, as the birds had to combine their knowledge of what they were looking for, where they had stored it, and when they stored it there, indicating that this was a complete memory for a unique event [16,17].

One challenge for the study of what–where–when memory in birds is that it relies on a heavily specialized adaptive capacity that evolved in some species subject to seasonal fluctuations in food supply; that of caching food for retrieval at a later point. While this does not preclude episodic memory from being used, it does open up the possibility that these animals have specialized forms of non-episodic memory to maximize the efficiency of this behaviour [15]. It is therefore beneficial to have an episodic memory task in animals that relies on a more generalized and spontaneous behaviour where there is little, if any, explicit reward for particular behaviours, and therefore, the evolution of specific strategies for this particular behaviour is unlikely. The lack of explicit learning in these tasks also take advantage of incidental encoding, which is thought to be critical to episodic memory [18–20]. Taking advantage of animals’ innate and spontaneous preference to preferentially explore novel objects over familiar ones [21], Eacott & Norman [22] demonstrated how such tasks could be adapted to demonstrate memory for a specific, unique event.

**Figure 1.**
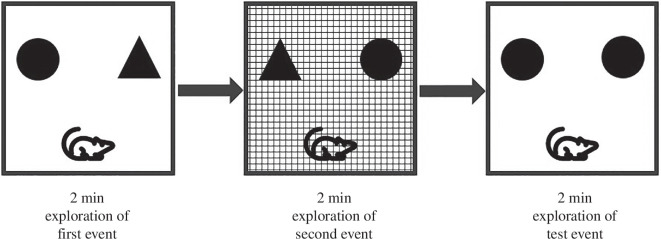
Example of what–where–which occasion task in rats. Animals explore one context (plain arena) for 2 min with objects placed to the left and right. After 2 min, animals are taken out and then returned to the same arena but with a new context inserted (checked pattern) with copies of the objects in swapped positions. After a further 2 min exploration, animals are removed for a delay period before being returned to one of the previously seen contexts (in this example, the plain context seen first) with two copies of just one of the objects present (in this case the circle) in both the left and right positions. In this case, the combination of the circle on the right in the plain context is a novel combination, and preferential exploration of this object serves as a behavioural indicator of the previous events.

In Eacott & Norman’s task [22], animals spontaneously explore (without explicit reward) an arena in which there are two objects (e.g. figure 1, circle and triangle) in two specific locations (left and right) in a specific visual-tactile context (a wall and floor insert in the arena; plain in figure 1). After 2 min of exploring, they are removed from the arena before being put on their own in a separate holding cage for a short (2 min) delay. After this delay, they are returned to the arena but now with a new context present (checked pattern in figure 1), and two copies of the same objects (circle and triangle), but now in swapped locations (i.e. in figure 1, the circle appears on the left in the first, plain, context, but then appears on the right in the second, patterned, context). After a second delay, the animals are replaced into one of the previous contexts (e.g. in figure 1, they return to the first, plain, context) with a copy of one of the objects (e.g. circle) in both the left and right positions. Now objects, their locations, and the contexts, they occur in are not novel to the animal. What is novel, however, is the unique combination of (in this case) the circle on the right, as the circle has never previously been seen on the right in this plain context. Therefore, preferential exploration of the circle on the right (in this example) over the circle on the left shows the animal can identify novelty based on what object is in what location in a particular context. To be able to identify this novelty requires a memory of the previous occasion of seeing the plain context, but where in that context the circle was only on the left, and the triangle on the right. Therefore, the preference for the novel combination in this task demonstrates a memory for what was where on which specific occasion, or what–where–which occasion memory [22,23]. As for the temporal cue in the scrub jay task [16], the purpose of context here is to cue the animal to remember one specific, and unique, event from its many previous experiences.

## 3. Comparing humans and non-human animals on measures of episodic memory

From the perspective of episodic memory, the what–where–when and what–where–which occasion tasks provide evidence for animals to use the content of the memory to influence behaviour. However, for MTT, they offer limited insight. Memory for a specific event need not require projection into that previous event as part of the remembering process. In humans, however, this can be tested by remember/know paradigms. Here participants are presented with lists of stimuli to be remembered. They are then given a memory test including some items from the original list and asked whether specific items are old or new. If they report having seen an item in the initial list (old response), they are then asked whether they remember or just know that to be the case [24]. Remember responses are defined as containing an explicit source of the memory (incidental features from the time of encoding, or some specific aspect of the experience of encoding). By contrast, know responses are source free, that is, they have a knowledge that they have seen it before but cannot recall elements of the experience of encoding (e.g. [25,26]). In these examples, remember responses reflect knowledge of source information which might reflect participants mentally travelling in time to situate themselves in their memory for the event.

**Figure 2.**
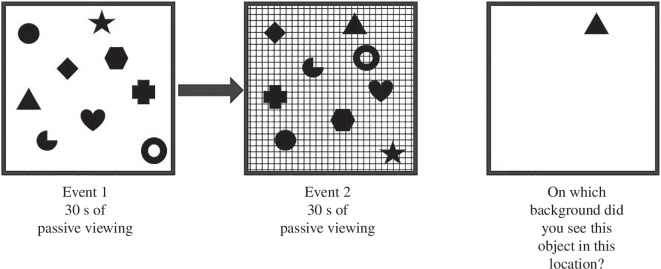
Participants passively viewed a screen with nine different objects in nine different positions for 30 s as event 1. There was no explicit instruction to remember the scene. After a short delay a second event was passively viewed for a further 30 s. On this screen, the same objects were presented in swapped locations on a new background. At test, what–where–which memory was assessed by asking participants to recall (by use of selecting one of the backgrounds presented visually) the correct background against which the seen object had been presented in the seen position.

Mirroring the content-based tasks of episodic memory in birds and rats, Easton *et al.* [27] asked human participants to look at arrangements of objects against specific backgrounds without explicitly being asked to remember anything about these arrangements (figure 2). Participants then report whether they remembered or knew the answers from memory. When participants were asked to report which background context an object had been seen in a particular location (what–where–which occasion memory) performance was only above chance when participants demonstrated that they remembered the event of encoding, i.e. they remembered source information from the encoding event. This supports the notion that this form of content-based task is primarily accessing source memory which might reflect the use of MTT. By contrast, when participants were shown the same types of objects but against a plain background and asked to report whether they saw it as part of the first or second event (like the what–where–when task of [16] where time is used as an indicator of how long ago the memory occurred) participants were above chance whether they used to remember or know strategies to answer the question. Therefore, although source memory was sometimes reported in this what–where–when task, that was not always the case. Participants who could not remember any source information from the encoding event were still able to perform the what–where–when task. Given that MTT requires memory for source information to reconstruct events this suggests that what–where–when memory does not necessarily involve MTT. These findings have been replicated in an immersive virtual environment reinforcing the finding that while context-based memories can only be remembered using source information, time-based memory does not necessarily involve remembering specific sources. Again, this is consistent with context based memories always involving mentally travelling in time while time based memories can be supported using strategies that do not involve MTT [28].

The dissociation between two tasks apparently measuring the same episodic memory is not unique to humans. Davis *et al.* [29] examined performance on both types of memory as spontaneous recognition tasks in mice. While normal mice could complete both episodic tasks easily, animals with a triple genetic modification to express Alzheimer’s disease pathology in an age-dependent manner showed impaired performance on the what–where–which occasion task even while the same animals showed normal performance on the what–where–when task. Similarly, what–where–which occasion tasks are shown to be more strongly affected by sleep than what–where–when tasks in rats [30].

What is critical from these studies is not that what–where–when is a poor model of episodic memory (it is not, and is fully a part of the broader definition of episodic memory used in what–where–which occasion memory). Instead, it is that when interpreting behaviour as a measure of putative cognitive processes, care must be taken to distinguish between alternative possibilities for what cognitive processes are involved. Easton *et al.* [27] argue that in these particular what–where–when conditions an alternative solution is available to participants. People may be able to place the object in a location to the correct place in time (first or second event) not by mentally time travelling to the point of encoding, but by introspecting the strength of the memory trace for the knowledge that they have without such MTT. For time (but not context), a simple assumption can be made; weak memory traces will be associated with the event longer ago in time, and stronger memory traces with the event closer in time. Like any assumption, this will not always be accurate, but it allows a route with enough accuracy to give participants above chance levels of performance without having to mentally travel in time to the event being remembered.

## 4. Distinguishing episodic memory from mental time travel

It could be argued that this is a particular failing of content-based tasks of episodic memory; the absence of a requirement to demonstrate MTT as part of the memory allows non-episodic solutions to be successful. However, to that end, it is worth considering the types of tasks commonly used to assess episodic memory in humans. For example, word list learning (e.g. [31]) or retelling of a story (e.g. [32]) are commonly used as measures of episodic memory. While in tasks such as word list learning, remember/know responses can be applied allowing some insight into whether participants have mentally travelled in time back to the memory being recalled, this is not always done. The possibility of being able to use episodic memory is often regarded as sufficient to call these measures of episodic memory. However, the findings from the what–where–when studies show that there are often multiple potential solution mechanisms available in behavioural tests of episodic memory and should guard against assumptions about which strategy is employed.

Critically, these assumptions about available strategies for solving behavioural tasks are not limited to those facing the past in episodic memory. In a common task of episodic future thinking (the spoon task; e.g. [33]) participants encounter a problem in one context, before needing to select from objects in a second context to find a solution to the problem. Children as young as 4 years old can pass this test [33], and the ability has also been demonstrated in apes [34] and corvids [35]. However, Dickerson *et al.* [36] evaluated a solution which produced the correct behaviour but through associative, rather than MTT, means. When the items being chosen between were both associated with positive outcomes (but only one remained useful for solving the problem) then older children (aged 5 years and over) could still choose the appropriate object above chance, but younger children (aged 3 or 4 years) did not. It appears, then, that some behavioural tasks designed to evaluate future episodic thinking have potential solutions that do not always, or only, require mental projection into the future.

We would argue that the great challenge set to those working on episodic memory in non-human species has been to demonstrate MTT in species who cannot communicate to us their inner experience. However, while this is sometimes presented as a failing of this work in animals (e.g. [2]), we see the same lack of MTT demonstration in much human work, even though some of these tasks clearly have solutions that do not require MTT.

If MTT is not always checked, even in common human tasks, and its use or otherwise sometimes has no effect on the memory performance, to what extent is MTT really a critical element of episodic memory?

One approach to this question is to consider the nature of MTT in those with aphantasia. Aphantasia describes those who have no visual mental imagery and evidence suggests these individuals have some difficulty in providing rich detail in their episodic memories (e.g. [37]). Nonetheless, Aydin [38] shows some evidence that this re-experiencing of the event can be dissociated from the memory for the event itself, and that the relationship between imagery and the details of the event reconstruction are true for memories of the past, but not for imagined future events. What is clearly true, however, is that the absence of mental imagery and a reduction in the quality of the experience when projecting into the event being remembered are less well linked to the accuracy of the memory itself. Of course, the past event can be constructed using non-visual imagery, but critically there seems to be a distinction between the experience of MTT and the accuracy of episodic memory.

Consider the alternative case of vicarious memories. When discussing episodic memory, it is critical in the definition that we discuss only memory for personally experienced events. By contrast, vicarious memories are recollections of events that have happened to others, whether they be a family member sharing a memory, or even a character in literature’s event as described in a book. Pillemer *et al.* [39] compared the experiential aspects of such vicarious memories with the memories for personally experienced events and found that while ratings of phenomenological characteristics of the memory were routinely higher for personally experienced rather than vicarious memories, the pattern of scores were similar, even when including vividness and imagery.

## 5. A specific role for episodic memory in mental time travel

Vicarious memories offer us, perhaps, an insight into the nature of MTT and its apparent dissociation from episodic memory. Why should we be able to have vivid imagery and similar phenomenology for remembering others’ memories as well as our own? What is the purpose of mental time travelling to another’s memories? Perhaps here it is worth reminding ourselves that the value of episodic memory (as for all types of memory) lies in future behaviour. A memory of eating toast for breakfast has little, if any, purpose other than to guide future actions (choosing what you eat next). Therefore, the value of others’ memories becomes obvious: others’ experiences can influence our future decisions as much as our own. If our ancestors encountered a story of someone being attacked by a bear outside a particular cave, it is important for that ancestor to avoid the cave, even though they personally did not witness or experience the attack.

However, the value of others’ memories might be clear, but this does not necessarily explain the need for MTT and experiences of revisiting others’ memories. It is, however, possible that MTT offers a particular advantage. While much ignored within neuroscience and experimental psychology studies of memory, 4E cognition (cognition that is embodied, embedded, enacted and extended, demonstrating an interaction between brain, behaviour, body and the environment) has become a widespread approach to cognition in other disciplines (see [40]). For the case of memory, 4E cognition is often considered in terms of how the memory is created or recalled. However, as we discussed above, the purpose of memory becomes apparent when making decisions for the future. One therefore might consider the cognitive processes around these decisions as fundamentally embodied, but ‘offline’ when not yet in the environment where the behavioural plan will be implemented (e.g. [41]). Consider the hungry person deciding what to eat. The memory of earlier eating toast is not the only factor in the decision. As discussed earlier, the environment also plays a part; the contents of the fridge provide limits to the choices available and therefore shape the possible options for lunch. The physiological consequences of having eaten toast may create an unconscious, somatic bias (literally, a gut feeling) towards preferring other options. The action of opening the fridge to look at its contents enacts the cognition and enables a decision informed by the environment. However, what if the person is sitting at work wondering what they will eat when they get home? The action of opening the fridge to consider the environmental limits of the options available cannot be used. However, the specific memory of the last time you opened the fridge (an episodic memory) allows us to internally simulate the environment in a way we can manipulate, interact with and explore. In this way, embodied simulation can be used to inform decisions.

A role for the hippocampus in constructing such manipulatable, spatially coherent, scenes can explain the structure’s involvement in memory, episodic future thinking and in predicting the future [42,43]. It offers the possibility that the purpose of MTT is not to remember a past event accurately, but rather to allow simulations of the real world (based on memory) that allow us to make better decisions about the future. This view is broadly supportive of recent perspectives that the hippocampus is not a memory structure *per se* but rather offers specific functions (such as construction of scenes) that support memory functions [42–44].

Studies of cellular mechanisms supporting memory within the hippocampus are also consistent with this suggestion. Initial studies showed that pyramidal cells in the hippocampus have clearly defined and persistent spatial firing patterns [45]. The combination of cells that represent different parts of environment can be used to support a cognitive map of an environment [46]. There have been hundreds of studies examining how and what aspects of the environment these ‘place cells’ represent [47,48]. Interestingly, the majority of these have assumed that this is a memory signal allowing an individual to internally represent previously experienced external space. However, in more recent years, there have been numerous examples of place cells representing not just the current location of the animal but also the future intended destination [49–53]. This would provide a cellular mechanism that would allow the hippocampus to evaluate future scenarios within a memory-guided framework.

We argue, therefore, that MTT is not synonymous with episodic memory as typically conceived. Tasks of episodic memory in animals do not, therefore, need to demonstrate MTT in order to be successful. Equally, MTT is not an irrelevance to episodic memory. Conceptualization of episodic memory and MTT in these ways produces positions which are fundamentally incompatible (e.g. [54]). Instead, MTT can allow improved behavioural choices based on episodic memory. Work in humans and non-human animals should be more comparable to ensure measures of memory are consistent, and generalizable, allowing work to bridge across species [55]. Careful consideration of behavioural outputs should be carried out in order to ensure that the cognitive mechanism under investigation is consistent with the measure being used. In addition, the unpicking of episodic memory from MTT opens up clear, testable questions about efficient, environmentally limited behaviours based on episodic memory.

## Data Availability

This article has no additional data.
